# Preditores de Terapias Apropriadas e Óbito em Pacientes com Cardiodesfibrilador Implantável e Cardiopatia Chagásica Crônica

**DOI:** 10.36660/abc.20230337

**Published:** 2024-07-23

**Authors:** Francisca Tatiana Moreira Pereira, Eduardo Arrais Rocha, Davi Sales Pereira Gondim, Rosa Livia Freitas de Almeida, Roberto da Justa Pires

**Affiliations:** 1 Universidade Federal do Ceará Fortaleza CE Brasil Universidade Federal do Ceará, Fortaleza, CE – Brasil; 2 Universidade de Fortaleza Fortaleza CE Brasil Universidade de Fortaleza, Fortaleza, CE – Brasil; 3 Tulane University School of Public Health and Tropical Medicine New Orleans Louisiana EUA Tulane University School of Public Health and Tropical Medicine, New Orleans, Louisiana – EUA; 4 Hospital São José de Doenças Infecciosas Fortaleza CE Brasil Hospital São José de Doenças Infecciosas, Fortaleza, CE – Brasil

**Keywords:** Doença de Chagas, Morte Súbita, Desfibriladores Implantáveis

## Abstract

**Fundamento:**

Existem poucos estudos retrospectivos e prospectivos sobre cardiodesfibrilador implantável (CDI) na prevenção primária e secundária de morte súbita na cardiopatia chagásica crônica (CCC).

**Objetivos:**

Descrever a evolução a longo prazo dos portadores de CCC com CDI e identificar e analisar os preditores de mortalidade e de terapia apropriada do dispositivo nessa população.

**Métodos:**

Trata-se de um estudo prospectivo histórico com 117 pacientes portadores de CDI e CCC. Dispositivos foram implantados de janeiro de 2003 a dezembro de 2021. Fatores preditores de terapias apropriadas e mortalidade a longo prazo foram identificados e analisados. O nível de significância estatística é de p < 0,05.

**Resultados:**

Pacientes (n = 117) tiveram mediana de seguimento de 61 meses (25 a 121 meses), sendo o gênero masculino (74%) predominante e a mediana de idade de 55 anos (48 a 64 anos). Houve 43,6% de choques apropriados, 26,5% de estimulação cardíaca antitaquicardia (ATP) e 51% de terapias apropriadas. Durante o seguimento, 46 pacientes (39,7%) foram a óbito. A mortalidade foi de 6,2% pessoas-ano (intervalo de confiança [IC] 95%: 4,6 a 8,3), com 2 mortes súbitas durante o seguimento. A prevenção secundária (hazard ratio [HR] 2.1; IC 95%: 1,1 a 4,3; p = 0,029) e a fração de ejeção menor que 30% (HR 1.8; IC 95%: 1,1 a 3,1; p < 0,05) foram preditores de terapias apropriadas. Escore de Rassi intermediário apresentou uma forte associação com ocorrência de ATP isoladamente (p = 0,015). A classe funcional IV (p = 0,007), fração de ejeção do ventrículo esquerdo < 30 (p = 0,010) e a idade maior que 75 anos (p = 0,042) foram preditores de mortalidade total.

**Conclusão:**

Os desfibriladores na CCC apresentaram elevada incidência de acionamento apropriado especialmente naqueles pacientes de prevenção secundária, fração de ejeção do ventrículo esquerdo baixa e escore de Rassi intermediário. Os pacientes com insuficiência cardíaca congestiva, classe funcional avançada e idade maior que 75 anos apresentaram elevada mortalidade.

**Figure:**
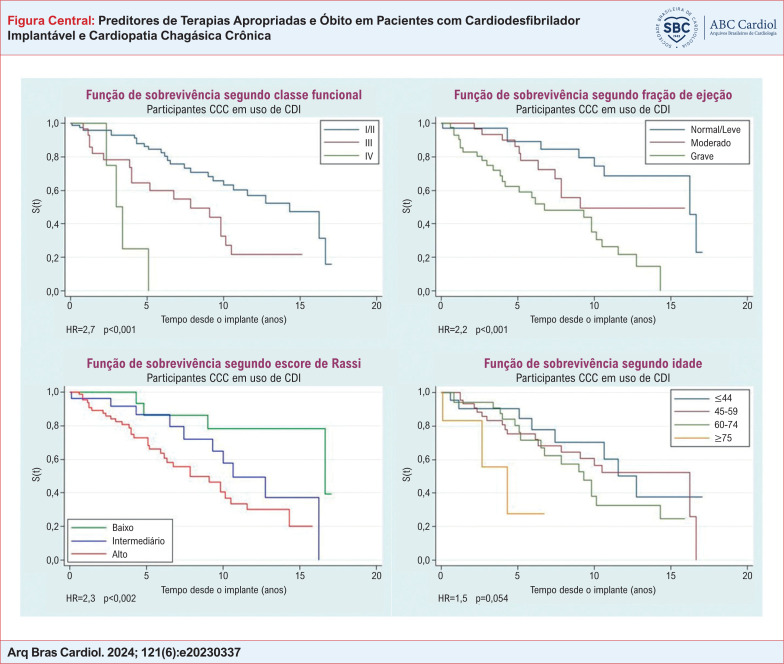
Função de sobrevivência dos pacientes com cardiodesfibrilador implantável e cardiopatia chagásica crônica. A - Segundo a classe funcional da New York Heart Association; B - Segundo a fração de ejeção do ventrículo esquerdo; C - Segundo escore de Rassi. D - Segundo a idade. CCC: cardiopatia chagásica crônica; CDI: cardiodesfibrilador implantável; HR: *hazard ratio* .

## Introdução

A doença de Chagas (tripanossomíase americana) é uma enfermidade causada pelo *Trypanosoma cruzi* , protozoário flagelado que infecta seres humanos, especialmente com maior vulnerabilidade social. Atualmente, a Organização Mundial da Saúde estima em aproximadamente 6 a 7 milhões o número de pessoas infectadas em todo o mundo, a maioria na América Latina. Em torno de 30% dos infectados podem evoluir para a forma cardíaca da doença que se caracteriza por ser uma cardiomiopatia dilatada inflamatória crônica. Essa, por sua vez, pode se manifestar clinicamente de várias formas: insuficiência cardíaca, arritmias, síncopes, fenômenos tromboembólicos e morte súbita cardíaca (MSC). O tratamento da cardiopatia chagásica crônica (CCC) se baseia na prevenção MSC e no controle dos sintomas, com medidas farmacológicas e não farmacológicas.^1^

A MSC por arritmia ventricular maligna (taquicardia ventricular sustentada [TVS] ou fibrilação ventricular [FV]) é responsável por 50% das mortes na CCC.^2^ Atualmente o cardiodesfibrilador implantável (CDI) é a principal estratégia de tratamento para evitar esses eventos. As evidências gerais da eficácia do CDI baseiam-se em ensaios clínicos de prevenção secundária (AVID, CASH e CIDS) e prevenção primária (MADIT I e II, MUSTT e SCD-HeFT). Esses estudos demonstraram a superioridade do CDI sobre os fármacos, em especial nas cardiomiopatias isquêmicas e idiopáticas.^3 – 8^

Existem poucos estudos retrospectivos e prospectivos sobre CDI na CCC. Desse modo, o presente estudo tem como objetivos descrever a evolução a longo prazo dos portadores de CCC com CDI e identificar os preditores de terapias apropriadas do dispositivo e de mortalidade nessa população.

## Métodos

Trata-se de um estudo de coorte prospectivo histórico, aprovado pelo Comitê de Ética em Pesquisa do Hospital Universitário Walter Cantídio da Universidade Federal do Ceará, no Brasil (protocolo: 4.165.388), realizado no período de janeiro de 2003 a dezembro de 2021.

Foram incluídos os pacientes maiores de 18 anos, de ambos os sexos, com diagnóstico de CCC que receberam CDI, com ou sem terapia de ressincronização cardíaca associada e que aceitaram participar do estudo. O trabalho foi realizado no serviço de estimulação cardíaca artificial do Hospital Walter Cantídio da Universidade Federal do Ceará durante o período de janeiro de 2003 a dezembro de 2021. As indicações para o implante de CDI estavam de acordo com as orientações das diretrizes brasileiras.^9 , 10^ Os pacientes que receberam o CDI para prevenção primária foram os com indicação para ressincronização e que não tinham apresentado previamente TVS ou MSC abortada por TVS ou FV.

Foram excluídos os pacientes menores de 18 anos ou que apresentaram concomitância de CCC e cardiopatia isquêmica. A doença coronariana foi excluída através do cateterismo cardíaco ou cintilografia miocárdica.

Um sistema de banco de dados foi projetado com as características clínicas e epidemiológicas dos pacientes, as indicações e os dados do CDI no momento do implante e durante o acompanhamento. Essas informações foram coletadas dos prontuários e durante consultas clínicas.

A programação do CDI incluía terapia com estimulação cardíaca antitaquicardia (ATP, do inglês, *antitachycardia pacing* ), otimizada para cada paciente, seguida de choque para TVS ou FV. Foi programada terapia inicial com ATP para TVS na presença de taquicardias com intervalo de ciclo variando entre 300 e 400 ms, com maior número de batimentos para detecção possível ou maior tempo de detecção, seguindo as diretrizes específicas. As programações de ATP foram com pelo menos 5 ATPs, tipo *bursts* , além de acionamento de todos os algoritmos discriminatórios para evitar acionamento inapropriado (choques inapropriados ou ATP inapropriados). As programações tipo rampa foram evitadas quando possível. O paciente avaliado que apresentasse no mesmo episódio várias tentativas de reversão por ATP era contabilizado com um episódio de terapia do dispositivo.

Considerou-se FV quando o ciclo do intervalo da taquicardia era inferior a 300 ms, sendo programados choques máximos após uma tentativa de reversão com ATP. Considerou-se TVS na presença de taquicardia sustentada com intervalo de ciclo variando entre 300 e 400 ms, não discriminado com taquicardia supraventricular por algoritmos específicos. Pacientes em uso de amiodarona que apresentavam redução da frequência cardíaca da TVS eram reprogramados com zonas exclusivamente de ATP para as frequências mais baixas, entre 120 e 150 bpm (400 e 500 ms) e com o maior número de ATP.

O choque ou ATP foram classificados apropriados para TVS/FV se o eletrograma intracardíaco gravado para intervenção fosse compatível. O choque ou ATP foram considerados inapropriados quando aplicados na ausência de TVS ou FV. Terapia apropriada consistiu em choque e/ou ATP apropriados.

O protocolo de acompanhamento incluiu consultas clínicas regulares e avaliação do dispositivo três vezes ao ano ou com prazos menores, conforme o número de terapias e a gravidade do paciente.

As análises por telemetria dos dispositivos e o acompanhamento desses pacientes foram realizados por três especialistas em estimulação cardíaca artificial do serviço. Em relação às dúvidas nas análises dos traçados ou na programação, outro especialista do serviço era acionado para avaliação.

Os pacientes com tempestades elétricas (três ou mais episódios de TVS/FV nas últimas 24 horas) foram internados para terapia farmacológica antiarrítmica venosa e o CDI reprogramado com otimização dos seus parâmetros. Quando persistiam os eventos arrítmicos, os pacientes eram encaminhados para o setor de eletrofisiologia e avaliados para ablação.

A função sistólica do ventrículo esquerdo pode ser avaliada através da medida da fração de ejeção do ventrículo esquerdo (FEVE) pelo ecocardiograma. Disfunção ventricular sistólica leve foi definida como FEVE entre 45% e 55%, disfunção moderada como FEVE entre 30% e 44%, e disfunção severa como FEVE < 30%. Função sistólica foi considerada normal quando a FEVE foi igual ou superior a 55%.

Os fatores preditores de terapias apropriadas (choque e/ou ATP) e mortalidade a longo prazo foram identificados e analisados. As variáveis coletadas foram as sociodemográficas (idade, sexo, nível de instrução e renda familiar) e clínicas (tipo de prevenção, FEVE, classe funcional, tipo de dispositivos, escore de Rassi e complicações do implante), selecionadas em conformidade com outros estudos realizados que evidenciaram possíveis associações de predição.^11 – 13^

As circunstâncias de morte foram classificadas a partir de uma causa cardíaca e não cardíaca, e a classificação de Hinkle e Thaler foi utilizada para avaliar a suspeita do mecanismo de morte.^14^

### Análise estatística

Os dados foram inseridos no REDCap e analisados no software SPSS, versão 25.0 e STATA 16. O teste de Shapiro-Wilk foi utilizado para aferir a normalidade das variáveis numéricas. Aquelas com distribuições normais foram descritas através de média ± desvio-padrão e as que não exibiram distribuição normal foram apresentadas através de mediana e intervalo interquartil.

Utilizou-se o teste qui-quadrado e exato de Fisher quando apropriado para comparações das variáveis categóricas, com apresentação de tabelas com valor absoluto (n) e relativo (%). Construíram-se curvas de Kaplan-Meier para variáveis com p < 0,05 e suas comparações foram realizadas com o teste log-rank bicaudal entre estratos. O nível de significância estatística é de p < 0,05.

A sobrevida cumulativa foi avaliada pelo método de regressão de Cox, ajustada para variáveis independentes, e as diferenças foram comparadas por modelos de riscos proporcionais de Cox. O estimador de Nelson-Aalen foi utilizado para determinar a probabilidade dos eventos de interesse.

Para avaliar a proporcionalidade do risco associado aos preditores, utilizou-se o teste de Schoenfeld e a inspeção gráfica dos resíduos de Cox-Snell.

## Resultados

Foram incluídos 117 pacientes com mediana de seguimento de 61 meses (25 a 121 meses), sendo o gênero masculino (74%) predominante e a mediana de idade de 55 anos (48 a 64 anos). As características epidemiológicas estão demonstradas na Tabela 1 . Os percentuais de alcoolismo, transfusão sanguínea e tabagismo foram 3,4%, 4,3% e 8,5%, respectivamente. Não houve relatos de uso de substâncias ilícitas. Havia 26% de hipertensos e 7% de diabéticos. Insuficiência renal crônica não dialítica estava presente em 3 pacientes. Nenhum com insuficiência renal dialítica.

**Tabela 1:** Característica basal da coorte em seguimento das pessoas acometidas com doença de Chagas com cardiodesfibrilador implantável

Características		(n=117)	%	IC 95%
**Sexo**				
	Masculino	87	74,4	(65,9 - 81,6)
	Feminino	30	25,6	(18,4 - 34,1)
**Idade**				
	Mediana (Q25 Q75)	55(48 - 64)		
**Nível de instrução**	
	Sem alfabetização	26	22,2	(15,4 - 30,4)
	1° Grau	65	55,6	(46,5 - 64,3)
	2° Grau	22	18,8	(12,5 - 26,6)
	3° Grau	4	3,4	(1,2 - 7,9)
**Estado civil**				
	Com companheiro	99	84,6	(77,3 - 90,3)
	Sem companheiro	18	15,4	(9,7 - 22,7)
**Renda mensal familiar**				
	< 3 salários mínimos	94	80,3	(72,5 - 86,8)
	3 – 7salários mínimos	17	14,5	(9 - 21,7)
	> 7 salários mínimos	6	5,1	(2,2 - 10,3)
**Alcoolismo**				
	Sim	5	4,3	(1,6 - 9,1)
	Ex-etilista	4	3,4	(1,2 - 7,9)
**Tabagismo**				
	Sim	10	8,5	(4,5 - 14,6)
**Origem**				
	Capital	31	26,5	(19,1 - 35)
	Outros municípios	86	73,5	(65 - 80,9)
**Conhece o barbeiro**				
	Sim	92	78,6	(70,6 - 85,3)
**Morou em casa de taipa**				
	Nunca	61	52,1	(43,1 - 61)
	Mora ou já morou	56	47,9	(39 - 56,9)
**Diabetes mellitus**				
	Sim	8	6,8	(3,3 - 12,5)
**Dislipidemia**				
	Sim	10	8,5	(4,5 - 14,6)

IC: intervalo de confiança.

Noventa e sete (82,9%) pacientes apresentavam algum grau de insuficiência cardíaca no momento da primeira avaliação (classe II, III ou IV da New York Heart Association) e apenas 18 (15,4%) apresentaram função ventricular esquerda sistólica normal pelo ecocardiograma transtorácico ( Tabela 2 ). Os pacientes foram classificados no momento da primeira consulta pelos critérios de Rassi e a maioria apresentava o valor alto (62,4%) ou intermediário de risco (22,2%). O bloqueio do ramo direito esteve presente no eletrocardiograma de 44 (37,6%) pacientes e o bloqueio do ramo esquerdo em 11 (9,4%) deles. Oitenta e quatro (71,7%) pacientes faziam uso do fármaco betabloqueador associado à amiodarona. Noventa e nove (84,6%) pacientes usavam amiodarona.

**Tabela 2:** Variáveis clínicas

Características clínicas		n=117	%	IC 95%
**Nível de prevenção**				
	Primária	30	25,6	18,4 - 34,1
	Secundária	87	74,4	65,9 - 81,6
**FEVE**				
	Normal	18	15,4	9,7-22,7
	Leve	16	13,7	8,4-20,8
	Moderada	38	32,5	24,5-41,3
	Severa	45	38,5	30-47,5
**CF pré-implante**				
	I	20	17,1	11,1-24,7
	II	61	52,1	43,1-61,0
	III	29	24,8	17,6-33,2
	IV	7	6	2,7-11,4
**Escore de Rassi**				
	Baixo risco	18	15,4	9,7-22,7
	Intermediário	26	22,2	15,4-30,4
	Alto risco	73	62,4	53,4-70,8
**Tipo dispositivo**				
	CDI-SR	8	6,8	3,3 - 12,5
	CDI-DR	86	73,5	65,0 - 80,9
	CRT-D	23	19,7	13,2 - 27,5
**Complicações no implante**				
	Sim	6	5,1	2,2 - 10,3
**Pneumotórax**				
	Sim	2	1,7	0,4 - 5,4
**Deslocamento do eletrodo**				
	Sim	3	2,6	0,7 - 6,7
**Óbito**				
	Sim	46	39,7	31,1 - 48,7
**Causas de óbito**				
	ICC refratária	27	58,7	44,3 - 72
	Tempestade elétrica	6	13,0	5,6 - 24,9
	Morte súbita	2	4,3	0,9 - 13,2
	AVC	1	2,2	0,2 - 9,7
	Outras causas	10	21,7	11,8 - 35,1

AVC: acidente vascular cerebral; CDI: cardiodesfibrilador implantável; CDI-DR: cardiodesfibrilador implantável dupla-câmara; CDI-SR: cardiodesfibrilador implantável de câmara única; CRT-D: desfibrilador de terapia de ressincronização cardíaca; CF: classe funcional da New York Heart Association; FEVE: fração de ejeção do ventrículo esquerdo; ICC: insuficiência cardíaca congestiva.

Síncope (75,4%) e dispneia (41,9%) foram os sintomas mais prevalentes na coorte. A taquicardia ventricular não-sustentada no Holter de 24 horas esteve presente em 113 participantes (96,6%). Em 87 indivíduos pertencentes à coorte (74%), a indicação do CDI foi a prevenção secundária de morte súbita e 6,1% evoluíram para transplante cardíaco após implante de CDI. O CDI dupla-câmara foi implantado em 73,5% dos pacientes. Não ocorreu óbito cirúrgico e a taxa de complicações dos implantes foi de 5,1% ( Tabela 2 ).

A incidência de choques apropriados e ATP foram 45,3% e 26,5%, respectivamente. A incidência de terapias apropriadas foi de 51,2%. O número de choques apropriados foi 338 (6,3 ± 1 por indivíduo). O número de ATP foi 190 (6,1 ± 1,8 por indivíduo). A taxa de choques inapropriados foi de 7,7%. Tempestade elétrica esteve presente em 26 pacientes (22,2%), e desses, 6 foram a óbito (23%). Nível de prevenção teve associação com terapia apropriada (p = 0,007) ( Tabela 6 ).

Durante o seguimento, 46 pacientes (39,7%) foram a óbito. A principal causa de morte foi a insuficiência cardíaca refratária (58,7%) seguida por causa não cardíaca em 21,7% dos pacientes. A mortalidade anual foi de 6,2% pessoas-ano (intervalo de confiança de 95%: 4,6 a 8,3), com apenas 2 mortes súbitas durante o seguimento. FEVE (p = 0,007) e classe funcional (p = 0,005) tiveram associação com óbito ( Tabela 7 ). A sobrevida ao óbito nos primeiros 5 anos de estudo foi de 77%, aproximadamente um risco acumulado pela estimativa de Nelson-Aalen de 26,1%.

A prevenção secundária e a FEVE menor que 30% foram preditores de terapias apropriadas ( Tabela 3 ).

**Tabela 3:** Preditores de terapias apropriadas na coorte de 117 pacientes

Fatores		Total n(%)	Terapia apropriada		
			Sim n(%)	Hz bruto (IC 95%)	Hz ajustado (IC 95%)
**Sexo**					
	Masculino	87 (74,4)	40 (46,0)	1	
	Feminino	30 (25,6)	20 (66,7)	1,6 (0,9 - 2,8)	
**Idade**					
	mediana (Q25-Q75)	55 (48 - 64)	54 (48 - 63,5)	1,0 (0,9 - 1,0)	1,0 (0,9 - 1,0)
**Prevenção**					
	Primária	30 (25,6)	9 (30,0)		
	Secundária	87 (74,4)	51 (58,6)	1,8 (0,9 - 3,7)	2,1 (1,1 - 4,3)
**Classe funcional**					
	I	20 (17,1)	11 (55)	1	
	II	61 (52,1)	31 (50,8)	0,7 (0,4 - 1,2)	
	III	29 (24,8)	16 (55,2)	1,3 (0,7 - 2,3)	
	IV	7 (6,0)	2 (28,6)	0,8 (0,2 - 3,3)	
**Fração de ejeção**				
	Normal	18 (15,4)	10 (55,6)	1	
	Leve	16 (13,7)	8 (50,0)	0,92 (0,4 - 1,92)	
	Moderada	38 (32,5)	18 (47,4)	0,6 (0,3 - 1,1)	
	Severa	45 (38,5)	24 (53,3)	1,5 (0,9 - 2,5)	1,8 (1,1 - 3,1)

Observou-se associação entre o escore de Rassi e a ocorrência de ATP apropriados (p = 0,015), com associação mais forte para o escore de Rassi intermediário (resíduo ajustado = 2,6) ( Tabela 4 ).

**Tabela 4:** Relação de terapias apropriadas da cardiodesfibrilador implantável e o escore de Rassi da coorte de 117 pacientes

		Escore de Rassi											p
		Total		Baixo risco			Intermediário			Alto risco			
		n	%	n	%	AR	n	%	AR	n	%	AR	
**Total**		117		18	15,4		26	22,2		73	62,4		
Choques													0,547
	Sim	62	53,0	10	16,1	0,2	16	25,8	1,0	36	58,1	–1,0	
**Choque apropriado**													0,342
	Sim	53	45,3	7	13,2	–0,6	15	28,3	1,4	31	58,5	–0,8	
**Choque inapropriado**													0,265
	Sim	9	7,7	3	33,3	1,6	1	11,1	–0,8	5	55,6	–0,4	
**ATP apropriado**													0,015
	Sim	31	26,5	6	19,4	0,7	12	38,7	2,6	13	41,9	–2,7	
**Terapia apropriada**													0,489
	Sim	60	51,3	9	15,0	–0,1	16	26,7	1,2	35	58,3	–0,9	
**Tempestade elétrica**													0,078
	Sim	26	22,2	3	11,5	–0,6	10	38,5	2,3	13	50,0	–1,5	

AR: resíduo ajustado; ATP: estimulação cardíaca antitaquicardia.

Na análise univariada de Cox, fração de ejeção < 30% (p < 0,001), escore de Rassi alto (p < 0,002) e classe funcional IV (p < 0,001) foram preditores de mortalidade total ( Figura Central ). O escore de risco de Rassi alto aumentou em 2,3 vezes o risco de óbito. A idade maior que 75 anos (p = 0,054) e o tipo de prevenção (p = 0,069) não tiveram significância estatística.

Na análise multivariada de Cox a classe funcional IV (p = 0,007), FEVE < 30 (p = 0,010) e idade maior 75 anos (p = 0,042) foram preditores de mortalidade total ( Tabela 5 ).

**Tabela 5:** Análise multivariada de Cox (mortalidade)

Fatores		HR	p	IC 96%	
**CF pré-imp**					
	I e II	1			
	III	1,5	0,211	0,8	3,1
	IV	5,4	0,007	1,6	18,5
FEVE pré-imp					
	Normal/leve	1,0			
	Moderada	1,8	0,250	0,7	4,7
	Severa	3,3	0,010	1,3	8,4
**Idade**					
	Acima de 75 anos	1,0	0,042	1,0	1,1

CF pré-imp: classe funcional pré-implante de cardiodesfibrilador implantável; FEVE pré-imp: fração de ejeção do ventrículo esquerdo pré-implante de cardiodesfibrilador implantável; HR: hazard ratio; IC: intervalo de confiança.

**Tabela 6:** Relação entre terapia apropriada e as variáveis sociodemográficas e clínicas na coorte de 117 pacientes em uso de cardiodesfibrilador implantável com cardiopatia crônica da doença de Chagas

Características		n(%)	Terapia apropriada		p
			Sim n(%)	Não n(%)	
**Idade (Q_25_ Q_75_)**		55 (48-64)	54 (48-63,5)	58 (48-64)	
**Sexo**					0,051
	Masculino	87(74,4)	40(46,0)	47(54)	
	Feminino	30(25,6)	20(66,7)	10(33,3)	
**Nível de instrução**					0,704
	Sem alfabetização	26(22,2)	15(57,7)	11(42,3)	
	1° grau	65(55,6)	34(52,3)	31(47,7)	
	2° grau	22(18,8)	9(40,9)	13(59,1)	
	3° grau	4(3,4)	2(50,0)	2(50,0)	
**Renda mensal familiar (SM)**					0,927
	< 3	94(80,3)	49(52,1)	45(47,9)	
	3 a 7	17(14,5)	8(47,1)	9(52,9)	
	> 7	6(5,1)	3(50,0)	3(50,0)	
**Nível de prevenção**					0,007
	Primária	30(25,6)	9(30)	21(70)	
	Secundária	87(74,4)	51(58,6)	36(41,4)	
**Fração de ejeção pré-implante**					0,93
	Normal	18(15,4)	10(55,6)	8(44,4)	
	Leve	16(13,7)	8(50,0)	8(50,0)	
	Moderada	38(32,5)	18(47,4)	20(52,6)	
	Severa	45(38,5)	24(53,3)	21(46,7)	
**Classe funcional pré-implante**				0,629
	I	20(17,1)	11(55)	9(45)	
	II	61(52,1)	31(50,8)	30(49,2)	
	III	29(24,8)	16(55,2)	13(44,8)	
	IV	7(6)	2(28,6)	5(71,4)	
**Dispositivo**					0,258
	CDI-SR	8(6,8)	2(25)	6(75)	
	CDI-DR	86(73,5)	47(54,7)	39(45,3)	
	CRT-D	23(19,7)	11(47,8)	12(52,2)	

CDI: cardiodesfibrilador implantável; CDI-DR: cardiodesfibrilador implantável dupla-câmara; CDI-SR: cardiodesfibrilador implantável de câmara única; CRT-D: desfibrilador de terapia de ressincronização cardíaca; SM: salários mínimos. Teste exato de Fisher/teste do qui-quadrado.

**Tabela 7:** Relação entre óbito e as variáveis sociodemográficas e clínicas na coorte de 117 pacientes em uso de CDI com cardiopatia crônica da doença de Chagas

Características		Total n(%)	Óbito		p
			Sim n(%)	Não n(%)	
**Idade**		56,47	58,30	55,30	
**Sexo**					0,929
	Masculino	87(74,4)	34(39,1)	53(60,9)	
	Feminino	30(25,6)	12(40)	18(60)	
**Nível de instrução**					0,668
	Sem alfabetização	26(22,2)	10(38,5)	16(61,5)	
	1° grau	65(55,6)	24(36,9)	41(63,1)	
	2° grau	22(18,8)	11(50)	11(50)	
	3° grau	4(3,4)	1(25)	3(75)	
**Renda mensal familiar (SM)**				71(60,7)	0,203
	< 3	94(80,3)	34(36,2)	60(63,8)	
	3 a 7	17(14,5)	10(58,8)	7(41,2)	
	> 7	6(5,1)	2(33,3)	4(66,7)	
**Nível de prevenção**					0,730
	Primária	30(25,6)	11(36,7)	19(63,3)	
	Secundária	87(74,4)	35(40,2)	52(59,8)	
**Fração de ejeção pré-implante**					0,007
	Normal	18(15,4)	3(16,7)	15(83,3)	
	Leve	16(13,7)	6(37,5)	10(62,5)	
	Moderada	38(32,5)	11(28,9)	27(71,1)	
	Severa	45(38,5)	26(57,8)	19(42,2)	
**Classe funcional pré-implante**				0,005
	I	20(17,1)	2(10)	18(90)	
	II	61(52,1)	23(37,7)	38(62,3)	
	III	29(24,8)	17(58,6)	12(41,4)	
	IV	7(6)	4(57,1)	3(42,9)	
**Dispositivo**					0,066
	CDI-SR	8(6,8)	1(12,5)	7(87,5)	
	CDI-DR	86(73,5)	32(37,2)	54(62,8)	
	CRT-D	23(19,7)	13(56,5)	10(43,5)	
**Complicações no implante**					0,582
	Sim	6(5,1)	3(50)	3(50)	

CDI: cardiodesfibrilador implantável; CDI-DR: cardiodesfibrilador implantável dupla-câmara; CDI-SR: cardiodesfibrilador implantável de câmara única; CRT-D: desfibrilador de terapia de ressincronização cardíaca; SM: salários mínimos. Teste exato de Fisher/teste do qui-quadrado.

## Discussão

No presente estudo, a mortalidade anual por todas as causas e a MSC tiveram taxas baixas, apesar da incidência elevada de choques, terapias e ATP apropriados, sugerindo a eficácia do CDI na prevenção primária e secundária de MSC na CCC, já que estudos com CCC sem CDI demonstraram uma incidência alta de morte súbita.^15 – 19^ Sarabanda et al. descreveram uma mortalidade anual de 10,7% em uma série de 28 pacientes sem CDI com seguimento médio 38 ± 16 meses, sendo 78% devido a morte súbita.^15^ Rassi et al. estudaram 424 pacientes com CCC durante um seguimento médio de 7,9 anos; eles encontraram uma taxa de 62% de morte súbita em pacientes sem CDI.^16^ Bestetti et al., estudando 74 pacientes com seguimento médio de 18 ± 12 meses, obtiveram taxa de 44% de MSC.^17^ Scanavacca et al. demonstraram uma incidência de 11% de MSC em uma coorte de 35 pacientes com TVS tratados com amiodarona seguidos por 3 anos.^18^ Mady et al. estudaram 104 pacientes com um seguimento médio de 30 ± 24 meses e encontraram uma taxa de 50% de mortalidade, sendo 64% devido à MSC.^19^

A taxa encontrada de acionamento apropriado (choques, terapias apropriadas e ATP) do CDI nesse estudo foi semelhante aos dados de outras séries.^12 , 20 – 22^ Martinelli et al. demostraram uma incidência de choque apropriado em 50% dos 116 pacientes com CCC e CDI por prevenção secundária de morte súbita no seguimento médio de 42 ± 32 meses.^12^ Barbosa demonstrou uma incidência de 62,7% de terapia apropriada em pacientes com CCC e CDI por prevenção secundária, durante um seguimento de 266 dias.^20^ Gali et al. em uma coorte de 76 pacientes com CDI por prevenção secundária de MSC e um seguimento médio de 33 ± 16 meses demonstraram que 72% tiveram acionamento apropriado do dispositivo e uma mortalidade anual de 4,8%.^21^ Pavão et al. encontraram, no seu estudo com 111 pacientes, uma taxa de acionamento apropriado do CDI de 72% com uma taxa de mortalidade anual de 8,4%.^22^

A taxa de choques maior do que ATP no nosso estudo pode decorrer da metodologia de contagem usada em que os pacientes com ATP seguidos de choques (no mesmo episódio) eram contabilizados apenas como choque, e pacientes com vários ATPs no mesmo episódio foram contabilizados como apenas 1 episódio de reversão por ATP.

Em 2018 foi publicada uma revisão sistemática e metanálise de seis estudos observacionais de 598 pacientes para avaliar a mortalidade por todas as causas em pacientes com CCC e prevenção secundária tratados com CDI (quatro estudos), amiodarona (um estudo) ou ambos (um estudo com duas coortes independentes, um tratado com CDI e outro tratado com amiodarona). Identificou-se que a mortalidade anual na população com CDI foi 9,7% versus 9,6% no grupo da amiodarona.^23^ Em 2019, outra revisão sistemática e metanálise mais robusta de 13 estudos observacionais de pacientes com CCC e CDI foi publicada. Foram incluídos 1041 pacientes, 8% de prevenção primária e 92% de secundária. A taxa de mortalidade total foi de 9% ao ano, e a taxa de MSC foi de 2% ao ano, em seguimento de 2,6 anos. Acionamento apropriado do CDI (choques ou terapias apropriadas) ocorreu em 24,8% dos pacientes, anualmente.^24^

Neste estudo, por ter seguimento mais prolongado, observou-se uma taxa de acionamento apropriado do CDI mais elevada, porém com uma mortalidade anual menor. O fato não pode ser explicado pela menor prevalência de prevenção secundária em relação às metanálises, já que o número de intervenções apropriadas pelo CDI neste estudo foi elevado. A possibilidade de terapias com ablação de arritmias ventriculares e transplante cardíaco para os casos graves com tempestades elétricas recorrentes ou insuficiência cardíaca congestiva refratária, o seguimento com especialistas, consultas ambulatoriais trimestrais e as constantes reprogramações dos dispositivos desses pacientes, podem ter contribuído para uma mortalidade menor.

Os portadores de CDI por prevenção secundária tiveram mais eventos apropriados do que os de prevenção primária, confirmando os achados já descritos de que pacientes com arritmias ventriculares documentadas previamente aumentam a chance de recorrência.

A FEVE menor que 30% foi preditora de acionamento apropriado do CDI neste estudo. Acredita-se que o miocárdio mais comprometido apresenta maior substrato arritmogênico, apesar de saber-se que a morte súbita por arritmias pode ocorrer mesmo em paciente com FEVE preservada, reforçando a natureza complexa e grave dessa patologia.^25 , 26^

O escore de Rassi intermediário apresentou associação com a ocorrência de ATP pelo CDI, sugerindo que esse talvez seja o grupo em que o CDI traria maior benefício. Já o escore de Rassi alto aumentou o risco de óbito apesar da presença do CDI.

A FEVE < 30%, classe funcional IV e idade acima de 75 anos na CCC foram preditoras de maior mortalidade. Esses achados foram semelhantes aos encontrados em outros estudos com CCC e CDI.^11 , 13^

## Conclusão

Os desfibriladores, quando utilizados em portadores de cardiopatia chagásica apresentaram elevada incidência de terapias apropriadas, baixa incidência de morte súbita e baixa taxa de terapias inapropriadas ou complicações em seguimento prolongado, sugerindo a eficácia desse tratamento para os grupos com alto risco de morte súbita, especialmente naquele grupo com escore de Rassi intermediário e prevenção secundária. Os pacientes com disfunção ventricular esquerda grave, idade acima de 75 anos e classe funcional avançada apresentaram elevada mortalidade.
