# Supplementary material for: Preditores de Terapias Apropriadas e Óbito em Pacientes com Cardiodesfibrilador Implantável e Cardiopatia Chagásica Crônica
Source: Arq Bras Cardiol. 2024 Jul 23;121(6):e20230337. [Article in Portuguese] doi: 10.36660/abc.20230337 (PMC11364444; doi:10.36660/abc.20230337)
Supplement: Translation

# Translation: Predictors of Appropriate Therapies and Death in Patients with Implantable Cardioverter-Defibrillator and Chronic Chagas Heart Disease

**DOI:** 10.36660/abc.20230337i

**Authors:** Francisca Tatiana Moreira Pereira, Eduardo Arrais Rocha, Davi Sales Pereira Gondim, Rosa Livia Freitas de Almeida, Roberto da Justa Pires

**Affiliations:** 1 Fortaleza CE Brazil Universidade Federal do Ceará, Fortaleza, CE – Brazil; 2 Fortaleza CE Brazil Universidade de Fortaleza, Fortaleza, CE – Brazil; 3 New Orleans Louisiana USA Tulane University School of Public Health and Tropical Medicine, New Orleans, Louisiana – USA; 4 Fortaleza CE Brazil Hospital São José de Doenças Infecciosas, Fortaleza, CE – Brazil

**Keywords:** Chagas Disease, Sudden Death, Implantable Defibrillators

## Abstract

**Background:**

There are few retrospective and prospective studies on implantable cardioverter-defibrillators (ICD) in primary and secondary prevention of sudden death in chronic Chagas heart disease (CCHD).

**Objectives:**

To describe the long-term evolution of patients with CCHD and ICD and to identify and analyze predictors of mortality and appropriate device therapy in this population.

**Methods:**

This was a historical prospective study with 117 patients with ICD and CCHD. Devices were implanted from January 2003 to December 2021. Predictors of appropriate therapies and long-term mortality were identified and analyzed. The level of statistical significance was p < 0.05.

**Results:**

Patients (n = 117) had a median follow-up of 61 months (25 to 121 months); they were predominantly male (74%), with a median age of 55 years (48 to 64 years). There were 43.6% appropriate shocks, 26.5% antitachycardia pacing (ATP), and 51% appropriate therapies. During follow-up, 46 patients (39.7%) died. Mortality was 6.2% person-years (95% confidence interval [CI]: 4.6 to 8.3), with 2 sudden deaths during follow-up. Secondary prevention (hazard ratio [HR] 2.1; 95% CI: 1.1 to 4.3; p = 0.029) and ejection fraction less than 30% (HR 1.8; 95% CI: 1.1 to 3.1; p < 0.05) were predictors of appropriate therapies. Intermediate Rassi score showed a strong association with the occurrence of ATP alone (p = 0.015). Functional class IV (p = 0.007), left ventricular ejection fraction < 30 (p = 0.010), and age above 75 years (p = 0.042) were predictors of total mortality.

**Conclusion:**

ICDs in CCHD showed a high incidence of appropriate activation, especially in patients with secondary prevention, low left ventricular ejection fraction, and intermediate Rassi score. Patients with congestive heart failure, elevated functional class, and age over 75 years showed elevated mortality.

**Figure:** Survival function of patients with implantable cardioverter-defibrillators and chronic Chagas heart disease. A – According to New York Heart Association functional class; B – According to left ventricular ejection fraction; C – According to Rassi score. D – According to age. CCHD: chronic Chagas heart disease; HR: hazard ratio; ICD: implantable cardioverter-defibrillator.

## Introduction

Chagas disease (American trypanosomiasis) is an illness caused by *Trypanosoma cruzi* , a flagellated protozoan that infects human beings, especially those with greater social vulnerability. Currently, the World Health Organization estimates that there are approximately 6 to 7 million people infected worldwide, the majority in Latin America. Around 30% of people who are infected can progress to the cardiac form of the disease, which is characterized as a chronic inflammatory dilated cardiomyopathy. This, in turn, can manifest clinically in different forms, including heart failure, arrhythmias, syncope, thromboembolic phenomena, and sudden cardiac death (SCD). The treatment of chronic Chagas heart disease (CCHD) is based on prevention of SCD and symptom control, with pharmacological and non-pharmacological measures.^1^

SCD due to malignant ventricular arrhythmias (sustained ventricular tachycardia [SVT] or ventricular fibrillation [VF]) is responsible for 50% of deaths in CCHD.^2^Implantable cardioverter-defibrillator (ICD) is currently the main treatment strategy to prevent these events. General evidence of ICD efficacy is based on clinical trials of secondary prevention (AVID, CASH, and CIDS) and primary prevention (MADIT I and II, MUSTT, and SCD-HeFT). These studies demonstrated the superiority of ICD over medications, especially in ischemic and idiopathic cardiomyopathies.^3 – 8^

There are few retrospective and prospective studies on ICD in CCHD. Therefore, the objectives of this study were to describe the long-term evolution of patients with CCHD and ICD and to identify predictors of appropriate device therapies and mortality in this population.

## Methods

This historical prospective cohort study received approval from the Research Ethics Committee of the Walter Cantídio University Hospital of the Universidade Federal do Ceará, in Brazil (protocol number: 4.165.388), and it was conducted during the period from January 2003 to December 2021.

The study included patients over 18 years of age, of both sexes, diagnosed with CCHD who received an ICD, with or without associated cardiac resynchronization therapy, and who agreed to participate in the study. The study was carried out at the artificial cardiac stimulation service of Walter Cantídio University Hospital da Universidade Federal do Ceará during the period from January 2003 to December 2021. The indications for ICD implantation were in accordance with the recommendations of Brazilian guidelines.^9 , 10^Patients who received an ICD for primary prevention had indication for resynchronization and had not previously presented with SVT or aborted SCD due to SVT or VF.

Patients under 18 years of age or those who presented concomitant CCHD and ischemic heart disease were excluded. Coronary heart disease was ruled out through cardiac catheterization or myocardial scintigraphy.

A database system was designed with patients’ clinical and epidemiological characteristics, indications and ICD data at the time of implantation and during follow-up. This information was collected from medical records and during clinical consultations.

ICD programming included antitachycardia pacing (ATP) therapy, optimized for each patient, followed by shock for SVT or VF. Initial ATP therapy was programmed for SVT in the presence of tachycardias with a cycle interval varying between 300 and 400 ms, with a greater number of beats for possible detection or a longer detection time, following specific guidelines. ATP was programmed with at least 5 ATP bursts, in addition to triggering all discriminatory algorithms to avoid inappropriate triggering (inappropriate shocks or inappropriate ATP). Ramp-type pacing was avoided when possible. Evaluated patients who presented several reversal attempts using ATP in the same episode were counted as one episode of device therapy.

VF was considered when the tachycardia cycle was less than 300 ms, with maximum shocks programmed after an attempted reversal with ATP. SVT was considered in the presence of sustained tachycardia with a cycle varying between 300 and 400 ms, not discriminated from supraventricular tachycardia by specific algorithms. Patients using amiodarone who had reduced SVT heart rate were reprogrammed with only ATP zones for the lowest frequencies, between 120 and 150 bpm (400 and 500 ms), with the highest number of ATP.

Shock or ATP was classified as appropriate for SVT/VF if the intracardiac electrogram recorded for intervention was compatible. Shock or ATP were considered inappropriate when applied in the absence of SVT or VF. Appropriate therapy consisted of appropriate shock and/or ATP.

The follow-up protocol included regular clinical consultations and device evaluation 3 times a year or in shorter intervals, depending on the number of therapies and the patient's degree of severity.

Telemetry analyses of the devices and patient monitoring were carried out by 3 specialists in artificial cardiac stimulation at the service. When there were doubts regarding the analysis of the tracings or the programming, another specialist from the service was invited for evaluation.

Patients with electrical storms (3 or more episodes of SVT/VF in the past 24 hours) were preferably hospitalized for intravenous antiarrhythmic pharmacological therapy, and the ICD was reprogrammed with optimization of parameters. When arrhythmic events persisted, patients were referred to the electrophysiology department and evaluated for ablation.

Left ventricular systolic function can be assessed by measuring left ventricular ejection fraction (LVEF) on echocardiography. Mild systolic ventricular dysfunction was defined as LVEF between 45% and 55%, moderate dysfunction as LVEF between 30% and 44%, and severe dysfunction as LVEF < 30%. Systolic function was considered normal when LVEF was equal to or greater than 55%.

Predictors of appropriate therapies (shock and/or ATP) and long-term mortality were identified and analyzed. The variables collected were sociodemographic (age, sex, education level, and family income) and clinical (type of prevention, LVEF, functional class, device type, Rassi score, and implant complications), selected in accordance with other studies that highlighted possible predictive associations.^11 – 13^

Circumstances of death were classified based on cardiac or non-cardiac cause, and the Hinkle and Thaler classification was used to evaluate the suspected mechanism of death.^14^

### Statistical analysis

Data were entered into REDCap and analyzed using SPSS software, version 25.0 and STATA 16. The Shapiro-Wilk test was used to assess the normality of numerical variables. Variables with normal distributions were described using mean ± standard deviation, and those that did not show normal distribution were presented using median and interquartile range.

The chi-square and Fisher's exact tests were used when appropriate to compare categorical variables, displayed in tables with absolute (n) and relative (%) values. Kaplan-Meier curves were constructed for variables with p < 0.05, and comparisons were made using the two-tailed log-rank test between strata. The level of statistical significance was p < 0.05.

Cumulative survival was assessed using the Cox regression method, adjusted for independent variables, and differences were compared using Cox proportional hazards models. The Nelson-Aalen estimator was used to determine the probability of events of interest.

The Schoenfeld test and graphical inspection of Cox-Snell residuals were used to assess the proportionality of the risk associated with the predictors.

## Results

The study included 117 patients with a median follow-up of 61 months (25 to 121 months); the male sex was predominant (74%), and median age was 55 years (48 to 64 years). The epidemiological characteristics are displayed in Table 1 . The percentages of alcoholism, blood transfusion, and smoking were 3.4%, 4.3% and 8.5%, respectively. None reported use of illicit substances. There were 26% with hypertension and 7% with diabetes. Non-dialysis chronic kidney failure was present in 3 patients. None had kidney failure on dialysis.

**Table 1:** Baseline characteristics of the cohort in follow-up of patients affected by Chagas disease with implantable cardioverter-defibrillators

Characteristics		(n=117)	%	95% CI
**Sex**				
	Male	87	74.4	(65.9 - 81.6)
	Female	30	25.6	(18.4 - 34.1)
**Age**				
	Median (25th to 75th quartile)	55(48 - 64)		
**Level of education**				
	None	26	22.2	(15.4 - 30.4)
	Primary	65	55.6	(46.5 - 64.3)
	Secondary	22	18.8	(12.5 - 26.6)
	Tertiary	4	3.4	(1.2 - 7.9)
**Marital status**				
	In a relationship	99	84.6	(77.3 - 90.3)
	Single	18	15.4	(9.7 - 22.7)
**Monthly family income**				
	< 3 times minimum wage	94	80.3	(72.5 - 86.8)
	3 to 7 times minimum wage	17	14.5	(9 - 21.7)
	> 7 times minimum wage	6	5.1	(2.2 - 10.3)
**Alcoholism**				
	Yes	5	4.3	(1.6 - 9.1)
	Former alcoholism	4	3.4	(1.2 - 7.9)
**Tabacco use**				
	Yes	10	8.5	(4.5 - 14.6)
**Origin**				
	Capital city	31	26.5	(19.1 - 35)
	Other municipalities	86	73.5	(65 - 80.9)
**Familiar with the insect**				
	Yes	92	78.6	(70.6 - 85.3)
**Lived in a clay house**				
	Never	61	52.1	(43.1 - 61)
	Currently living or had lived	56	47.9	(39 - 56.9)
**Diabetes mellitus**				
	Yes	8	6.8	(3.3 - 12.5)
**Dyslipidemia**				
	Yes	10	8.5	(4.5 - 14.6)

CI: confidence interval.

At the time of the first evaluation, 97 (82.9%) patients had some degree of heart failure (New York Heart Association class II, III, or IV), and only 18 (15.4%) had normal left ventricular systolic function on transthoracic echocardiogram ( Table 2 ). At the time of the first consultation, patients were classified using the Rassi criteria, and the majority had high (62.4%) or intermediate risk (22.2%). Right bundle branch block was present on the electrocardiogram of 44 (37.6%) patients and left bundle branch block in 11 (9.4%). Eighty-four (71.7%) patients were using beta blockers associated with amiodarone, and 99 (84.6%) patients were using amiodarone.

**Table 2:** Clinical variables

Clinical characteristics		n=117	%	95% CI
**Level of prevention**				
	Primary	30	25,6	18,4 - 34,1
	Secondary	87	74,4	65,9 - 81,6
**LVEF**				
	Normal	18	15,4	9,7-22,7
	Mild	16	13,7	8,4-20,8
	Moderate	38	32,5	24,5-41,3
	Severe	45	38,5	30-47,5
**Pre-implantation FC**				
	I	20	17,1	11,1-24,7
	II	61	52,1	43,1-61,0
	III	29	24,8	17,6-33,2
	IV	7	6	2,7-11,4
**Rassi score**				
	Low risk	18	15,4	9,7-22,7
	Intermediate risk	26	22,2	15,4-30,4
	High risk	73	62,4	53,4-70,8
**Device type**				
	ICD-SR	8	6,8	3,3 - 12,5
	ICD-DR	86	73,5	65,0 - 80,9
	CRT-D	23	19,7	13,2 - 27,5
**Implantation complications**				
	Yes	6	5,1	2,2 - 10,3
**Pneumothorax**				
	Yes	2	1,7	0,4 - 5,4
**Electrode displacement**				
	Yes	3	2,6	0,7 - 6,7
**Death**				
	Yes	46	39,7	31,1 - 48,7
**Cause of death**				
	Refractory CHF	27	58,7	44,3 - 72
	Electrical storm	6	13,0	5,6 - 24,9
	Sudden death	2	4,3	0,9 - 13,2
	Stroke	1	2,2	0,2 - 9,7
	Other causes	10	21,7	11,8 - 35,1

CHF: congestive heart failure; CRT-D: cardiac resynchronization therapy defibrillator; FC: New York Heart Association functional class; ICD: implantable cardioverter-defibrillator; ICD-DR: dual-chamber implantable cardioverter-defibrillator; ICD-SR: single-chamber implantable cardioverter-defibrillator; LVEF: left ventricular ejection fraction.

Syncope (75.4%) and dyspnea (41.9%) were the most prevalent symptoms in the cohort. Non-sustained ventricular tachycardia on 24-hour Holter monitoring was present in 113 participants (96.6%). In 87 individuals from the cohort (74%), the indication for ICD was secondary prevention of sudden death, and 6.1% progressed to heart transplantation after ICD implantation. A dual-chamber ICD was implanted in 73.5% of patients. There were no surgical deaths, and the rate of implant complications was 5.1% ( Table 2 ).

The incidence of appropriate shocks and ATP were 45.3% and 26.5%, respectively. The incidence of appropriate therapies was 51.2%. The number of appropriate shocks was 338 (6.3 ± 1 per individual). The number of ATP was 190 (6.1 ± 1.8 per individual). The rate of inappropriate shocks was 7.7%. Electrical storm was present in 26 patients (22.2%), and 6 of them died (23%). Level of prevention was associated with appropriate therapy (p = 0.007) ( Table 6 ).

During follow-up, 46 patients (39.7%) died. The main cause of death was refractory heart failure (58.7%) followed by non-cardiac causes in 21.7% of patients. Annual mortality was 6.2% person-years (95% confidence interval: 4.6 to 8.3), with only 2 sudden deaths during follow-up. LVEF (p = 0.007) and functional class (p = 0.005) were associated with death ( Table 7 ). Survival from death in the first 5 years of the study was 77%, an approximate cumulative risk of 26.1% according to the Nelson-Aalen estimate.

Secondary prevention and LVEF below 30% were predictors of appropriate therapies ( Table 3 ).

**Table 3:** Predictors of appropriate therapy in the cohort of 117 patients

Factors		Total n(%)	Terapia apropriada		
			Yes n(%)	Unadjusted Hz (95% CI)	Adjusted Hz (95% CI)
**Sex**					
	Male	87 (74.4)	40 (46.0)	1	
	Female	30 (25.6)	20 (66.7)	1.6 (0.9 - 2.8)	
**Age**					
	Median (25th to 75th quartile)	55 (48 - 64)	54 (48 - 63.5)	1.0 (0.9 - 1.0)	1.0 (0.9 - 1.0)
**Prevention**					
	Primary	30 (25.6)	9 (30.0)		
	Secondary	87 (74.4)	51 (58.6)	1.8 (0.9 - 3.7)	2.1 (1.1 - 4.3)
**Functional class**					
	I	20 (17.1)	11 (55)	1	
	II	61 (52.1)	31 (50.8)	0.7 (0.4 - 1.2)	
	III	29 (24.8)	16 (55.2)	1.3 (0.7 - 2.3)	
	IV	7 (6.0)	2 (28.6)	0.8 (0.2 - 3.3)	
**Ejection fraction**					
	Normal	18 (15.4)	10 (55.6)	1	
	Mild	16 (13.7)	8 (50.0)	0.92 (0.4 - 1.92)	
	Moderate	38 (32.5)	18 (47.4)	0.6 (0.3 - 1.1)	
	Severe	45 (38.5)	24 (53.3)	1.5 (0.9 - 2.5)	1.8 (1.1 - 3.1)

An association was observed between Rassi score and the occurrence of appropriate ATP (p = 0.015), with a stronger association for intermediate Rassi score (adjusted residual = 2.6) ( Table 4 ).

**Table 4:** List of appropriate implantable cardioverter-defibrillator therapies and Rassi score in the cohort of 117 patients

		Rassi score											p
		Total		Low risk			Intermediate risk			High risk			
		n	%	n	%	AR	n	%	AR	n	%	AR	
**Total**		117		18	15.4		26	22.2		73	62.4		
**Shocks**													0.547
	Yes	62	53.0	10	16.1	0.2	16	25.8	1.0	36	58.1	–1.0	
**Appropriate shock**													0.342
	Yes	53	45.3	7	13.2	–0.6	15	28.3	1.4	31	58.5	–0.8	
**Inappropriate shock**													0.265
	Yes	9	7.7	3	33.3	1.6	1	11.1	–0.8	5	55.6	–0.4	
**Appropriate ATP**													0.015
	Yes	31	26.5	6	19.4	0.7	12	38.7	2.6	13	41.9	–2.7	
**Appropriate therapy**													0.489
	Yes	60	51.3	9	15.0	–0.1	16	26.7	1.2	35	58.3	–0.9	
**Electrical storm**													0.078
	Yes	26	22.2	3	11.5	–0.6	10	38.5	2.3	13	50.0	–1.5	

AR: adjusted residual; ATP: antitachycardia pacing.

In Cox univariate analysis, ejection fraction < 30% (p < 0.001), high Rassi score (p < 0.002), and functional class IV (p < 0.001) were predictors of total mortality ( Central Illustration ). High Rassi risk score increased the risk of death 2.3-fold. Age over 75 years (p = 0.054) and type of prevention (p = 0.069) were not statistically significant.

In the Cox multivariate analysis, functional class IV (p = 0.007), LVEF < 30 (p = 0.010), and age over 75 years (p = 0.042) were predictors of total mortality ( Table 5 ).

**Table 5:** Cox multivariate analysis (mortality)

Factors		HR	p	96% CI	
**Pre-imp FC**					
	I and II	1			
	III	1.5	0.211	0.8	3.1
	IV	5.4	0.007	1.6	18.5
**Pre-imp LVEF**					
	Normal/mild	1.0			
	Moderate	1.8	0.250	0.7	4.7
	Severe	3.3	0.010	1.3	8.4
**Age**					
	Over 75 years	1.0	0.042	1.0	1.1

CI: confidence interval; HR: hazard ratio; Pre-imp FC: New York Heart Association functional class before implantable cardioverter-defibrillator implantation; Pre-imp LVEF: left ventricular ejection fraction before implantable cardioverter-defibrillator implantation.

**Table 6:** Relation between appropriate therapy and sociodemographic and clinical variables in the cohort of 117 patients with chronic Chagas disease heart disease using ICD

Characteristics		n(%)	Appropriate therapy		p
			Yes n(%)	No n(%)	
Age (25th to 75th quartile)		55 (48-64)	54 (48-63.5)	58 (48-64)	
**Sex**					0.051
	Male	87(74.4)	40(46.0)	47(54)	
	Female	30(25.6)	20(66.7)	10(33.3)	
**Level of education**					0.704
	None	26(22.2)	15(57.7)	11(42.3)	
	Primary	65(55.6)	34(52.3)	31(47.7)	
	Secondary	22(18.8)	9(40.9)	13(59.1)	
	Tertiary	4(3.4)	2(50.0)	2(50.0)	
**Monthly family income (MW)**					0.927
	< 3	94(80.3)	49(52.1)	45(47.9)	
	3 to 7	17(14.5)	8(47.1)	9(52.9)	
	> 7	6(5.1)	3(50.0)	3(50.0)	
**Level of prevention**					0.007
	Primary	30(25.6)	9(30)	21(70)	
	Secondary	87(74.4)	51(58.6)	36(41.4)	
**Pre-implantation ejection fraction**					0.93
	Normal	18(15.4)	10(55.6)	8(44.4)	
	Mild	16(13.7)	8(50.0)	8(50.0)	
	Moderate	38(32.5)	18(47.4)	20(52.6)	
	Severe	45(38.5)	24(53.3)	21(46.7)	
**Pre-implantation functional class**					0.629
	I	20(17.1)	11(55)	9(45)	
	II	61(52.1)	31(50.8)	30(49.2)	
	III	29(24.8)	16(55.2)	13(44.8)	
	IV	7(6)	2(28.6)	5(71.4)	
**Device type**					0.258
	ICD-SR	8(6.8)	2(25)	6(75)	
	ICD-DR	86(73.5)	47(54.7)	39(45.3)	
	CRT-D	23(19.7)	11(47.8)	12(52.2)	

CRT-D: cardiac resynchronization therapy defibrillator; ICD: implantable cardioverter-defibrillator; ICD-DR: dual-chamber implantable cardioverter-defibrillator; ICD-SR: single-chamber implantable cardioverter-defibrillator; MW: times minimum wage. Fisher's exact test/chi-square test.

**Table 7:** Relation between death and sociodemographic and clinical variables in the cohort of 117 patients with chronic Chagas disease heart disease using ICD

Characteristics		Total n(%)	Death		p
			Yes n(%)	No n(%)	
**Age**		56.47	58.30	55.30	
**Sex**					0.929
	Male	87(74.4)	34(39.1)	53(60.9)	
	Female	30(25.6)	12(40)	18(60)	
**Level of education**					0.668
	None	26(22.2)	10(38.5)	16(61.5)	
	Primary	65(55.6)	24(36.9)	41(63.1)	
	Secondary	22(18.8)	11(50)	11(50)	
	Tertiary	4(3.4)	1(25)	3(75)	
**Monthly family income (MW)**				71(60.7)	0.203
	< 3	94(80.3)	34(36.2)	60(63.8)	
	3 to 7	17(14.5)	10(58.8)	7(41.2)	
	> 7	6(5.1)	2(33.3)	4(66.7)	
**Level of prevention**					0.730
	Primary	30(25.6)	11(36.7)	19(63.3)	
	Secondary	87(74.4)	35(40.2)	52(59.8)	
**Pre-implantation ejection fraction**					0.007
	Normal	18(15.4)	3(16.7)	15(83.3)	
	Mild	16(13.7)	6(37.5)	10(62.5)	
	Moderate	38(32.5)	11(28.9)	27(71.1)	
	Severe	45(38.5)	26(57.8)	19(42.2)	
**Pre-implantation functional class**					0.005
	I	20(17.1)	2(10)	18(90)	
	II	61(52.1)	23(37.7)	38(62.3)	
	III	29(24.8)	17(58.6)	12(41.4)	
	IV	7(6)	4(57.1)	3(42.9)	
**Device type**					0.066
	ICD-SR	8(6.8)	1(12.5)	7(87.5)	
	ICD-DR	86(73.5)	32(37.2)	54(62.8)	
	CRT-D	23(19.7)	13(56.5)	10(43.5)	
**Implantation complications**					0.582
	Yes	6(5.1)	3(50)	3(50)	

CRT-D: cardiac resynchronization therapy defibrillator; ICD: implantable cardioverter-defibrillator; ICD-DR: dual-chamber implantable cardioverter-defibrillator; ICD-SR: single-chamber implantable cardioverter-defibrillator; MW: times minimum wage. Fisher's exact test/chi-square test.

## Discussion

In the present study, annual all-cause mortality and SCD had low rates despite the high incidence of appropriate shocks, therapies, and ATP, suggesting the effectiveness of ICD in the primary and secondary prevention of SCD in CCHD, as studies with CCHD without ICD demonstrated a high incidence of sudden death.^15 – 19^Sarabanda et al. described an annual mortality of 10.7% in a series of 28 patients without ICD, with a mean follow-up of 38 ± 16 months, and 78% were due to sudden death.^15^Rassi et al. studied 424 patients with CCHD during a mean follow-up of 7.9 years; they found a 62% rate of sudden death in patients without ICD.^16^Bestetti et al., studying 74 patients with a mean follow-up of 18 ± 12 months, obtained a SCD rate of 44%.^17^Scanavacca et al. demonstrated an incidence of 11% for SCD in a cohort of 35 patients with SVT treated with amiodarone followed for 3 years.^18^Mady et al. studied 104 patients with a mean follow-up of 30 ± 24 months and found a 50% mortality rate, 64% being due to SCD.^19^

The rate of appropriate ICD activation (shocks, appropriate therapies, and ATP) found in this study was similar to data from other series.^12 , 20 – 22^Martinelli et al. demonstrated an incidence of appropriate shock in 50% of 116 patients with CCHD and ICD for secondary prevention of sudden death in a mean follow-up of 42 ± 32 months.^12^Barbosa demonstrated a 62.7% incidence of appropriate therapy in patients with CCHD and ICD for secondary prevention, during a follow-up of 266 days.^20^Gali et al., in a cohort of 76 patients with ICD for secondary prevention of SCD and a mean follow-up of 33 ± 16 months, demonstrated that 72% had appropriate device activation, with an annual mortality of 4.8%.^21^Pavão et al. found, in their study with 111 patients, an appropriate ICD activation rate of 72% with an annual mortality rate of 8.4%.^22^

The rate of shocks was greater than that of ATP in our study; this may be due to the counting methodology used, in which patients with ATP followed by shocks (in the same episode) were counted only as shock, and patients with multiple ATPs in the same episode were counted as only 1 episode of reversal by ATP.

In 2018, a systematic review and meta-analysis was published with 6 observational studies of 598 patients to evaluate all-cause mortality in patients with CCHD and secondary prevention treated with ICD (4 studies), amiodarone (1 study), or both (1 study with 2 independent cohorts, one treated with ICD and the other treated with amiodarone). The study identified that annual mortality in the ICD population was 9.7% versus 9.6% in the amiodarone group.^23^In 2019, another more robust systematic review and meta-analysis was published, including 13 observational studies of patients with CCHD and ICD. The study included 1041 patients, 8% primary prevention and 92% secondary prevention. The total mortality rate was 9% per year, and the rate of SCD was 2% per year, with 2.6 years of follow-up. Appropriate ICD activation (shocks or appropriate therapies) occurred in 24.8% of patients annually.^24^

In this study, as it had longer follow-up, a higher rate of appropriate ICD activation was observed, but with lower annual mortality. This fact cannot be explained by the lower prevalence of secondary prevention in relation to meta-analyses, since the number of appropriate ICD interventions in this study was high. The possibility of therapies with ablation of ventricular arrhythmias and heart transplantation for severe cases with recurrent electrical storms or refractory congestive heart failure, follow-up with specialists, quarterly outpatient consultations, and constant reprogramming of these patients’ devices may have contributed to lower mortality.

Patients with ICD for secondary prevention had more appropriate events than those with ICD for primary prevention, confirming previously described findings that patients with prior documented ventricular arrhythmias have an increased chance of recurrence.

LVEF below 30% was a predictor of appropriate ICD activation in this study. It is believed that a more compromised myocardium has a greater arrhythmogenic substrate, although it is known that sudden death from arrhythmias can occur even in patients with preserved LVEF, reinforcing the complex and severe nature of this pathology.^25 , 26^

Intermediate Rassi score was associated with the occurrence of ATP by the ICD, suggesting that this may be the group in which the ICD would bring the greatest benefit. High Rassi score increased the risk of death notwithstanding the presence of the ICD.

LVEF < 30%, functional class IV, and age over 75 years in patients with CCHD were predictors of higher mortality. These findings were similar to those of other studies in patients with CCHD and ICD.^11 , 13^

## Conclusion

ICDs, when used in patients with CCHD, showed a high incidence of appropriate therapies, a low incidence of sudden death, and a low rate of inappropriate therapies or complications during prolonged follow-up, suggesting the effectiveness of this treatment for groups at high risk of sudden death, especially in the group with intermediate Rassi score and secondary prevention. Patients with severe left ventricular dysfunction, age over 75 years, and advanced functional class presented high mortality.
